# Supplementary material for: Are carbon emissions trading and green financial instruments synergistic? -Comprehensive quantitative research based on content analysis
Source: PLoS One. 2024 Mar 7;19(3):e0298601. doi: 10.1371/journal.pone.0298601 (PMC10919601; doi:10.1371/journal.pone.0298601)
Supplement: S1 Data — (ZIP) [file pone.0298601.s001.zip › Data/地级市Z_F工作报告.zip!/║╙─╧/σo╤⌠╩╨/└┤╫╘╣1⁄2╓┌║┼í╛┬φ┐╦╩2╛▌═°í┐_2003.docx]

各位代表：

现在，我代表市人民政府，向大会作工作报告，请予审议，并请市政协委员和其他列席人员提出意见。

一、2002年政府工作回顾

2002年，市人民政府在省委、省政府和市委的正确领导下，团结带领全市人民，以邓小平理论和“三个代表”重要思想为指导，紧紧围绕加快发展这一主题，大力实施“以工兴市”战略，克服困难，开拓进取，实现了经济持续发展和社会全面进步，顺利完成了市四届人大五次会议确定的目标任务。

国民经济快速增长。预计2002年，全市国内生产总值236.8亿元，比上年增长9.5％，较好地完成了“保9争10”的目标。其中第一产业增长4.9％，第二产业增长10.9％，第三产业增长10.1％。

农村经济稳定发展。去年7月初，黄河调水调沙我市滩区出险，在市委、市政府的坚强领导下，全市上下万众一心，奋力抢险护滩，把黄河漫滩造成的损失降到了最低限度，随后全力开展医疗防疫，妥善安排灾民生活，滩区大灾之后无大疫，使受灾群众达到了“有饭吃、有衣穿、有房住、有病及时治疗、儿童有学上”的“五有”要求。同时，战胜了持续干旱、冰雹等灾害的影响，全市粮食总产204.3万吨，虽比上年减产2%，但仍是历史上第三个丰收年。优质农作物面积进一步扩大，优质小麦151万亩、优质水稻30万亩，分别占其种植总面积的53%、63.6%。全市肉类总产18.54万吨、禽蛋总产22.9万吨、奶类总产0.92万吨，分别增长5.4%、7.9%和25.2%。生态建设取得新成绩，完成退耕还林5.11万亩，全市农田林网控制率90%，森林覆盖率18.5%。农田水利建设迈出新步伐，夺取省“红旗渠精神杯”市级杯三连冠。扶贫开发取得新进展，又有3.8万人脱贫。

工业速度效益同步增长。“以工兴市”战略成效显著。全市完成工业增加值105.91亿元，比上年增长10.5%，其中完成限额以上增加值79.64亿元，增长10％；销售收入190亿元，增长22％；产销率98.7%，比上年提高0.9个百分点；工业企业经济效益综合指数130.6%，居全省前列。市委、市政府重点抓的34个工业项目均完成和超额完成了建设任务，中原大化60万吨复合肥一期工程、濮耐公司连铸三大件生产线和500千伏输变电工程等建成投产，共完成投资41.92亿元，超计划22.93%。全市乡镇企业完成增加值50.6亿元，比上年增长10.3％；实现税金3.34亿元，增长7.7％。

第三产业快速发展。流通中心建设稳步推进，联华广场主楼裙楼、金桥家具广场等建成开业。全市社会消费品零售总额65.9亿元，比上年增长10.7％。旅游业有新的发展，绿色庄园被评为国家4A级景区，世锦园和毛楼生态旅游区被评为国家3A级景区。个体工商业从业人员7.58万人，注册资金2.82亿元，分别比上年增长17.7%和4%；私营企业2418户，注册资本14.8亿元，分别比上年增长19.3%和23%。

城市建设取得新进步。巩固创建成果，调整完善城市总体规划，强化城市基础设施建设和管理。东环路、黄河路中段、振兴路北段建成通车，皇甫国家粮库二期工程建成投用，市污水处理厂竣工，市人民医院外科综合楼和市中心血站业务楼主体完工，马颊河城区段治理工程、绿色庄园二期工程、濮水河治理二期工程、西环路改造工程、市体育场建设等进展顺利，新增供热面积12万平方米，新增经济适用住房面积19.42万平方米。在“四城一奖”的基础上，又荣获“迪拜国际改善居住环境良好范例奖”和“国际花园城市”铜牌奖。

重点项目建设实现重大突破。年初确定的87个重点项目和16件实事，除范县陆集移民建镇项目国家未予批复、黄淮海农业综合开发项目属跨年度工程没有完成外，其余均实现计划进度，共完成投资55.93亿元，超计划9.5%。特别是困扰我市多年的濮鹤高速公路项目，于去年4月18日正式开工并全线形成建设高潮；汤台铁路技术改造工程开工；林纸一体化项目具备了组织实施的条件。全市社会固定资产投资完成89.5亿元，比上年增长11.3％。其中地方完成45.7亿元，增长26.4％。各有关部门共争取国家、省各项资金17.7亿元，比上年增长14.2%。全市新建二级公路117公里，基本实现村村通油路。

改革开放取得新成绩。产权制度改革取得成效，全市国有及控股工业企业完成公司制改造21户，租赁经营19户，破产重组9户，其它形式改革12户，改制面66.3%。市面粉厂、密胺塑料厂成功实施破产、重组，现已恢复生产。市一机厂破产工作进入整体拍卖阶段。农村税费改革基本完成，亩均减负53.4%，人均减负53.3%。市县乡政府机构改革基本完成。市行政服务中心和财政集中收付中心投入运行，行政审批和财政体制改革稳步推进。经营城市成效显著，土地收益大幅增加。全市出口创汇3528万美元，比上年增长22.6%；外资实际到位3447万美元，增长3.2倍。市高新区引进项目92个，其中工业项目44个，引资总额4.2亿元；区内企业技工贸收入22亿元，比上年增长80%。工贸示范区新上项目102个，投资总额8.2亿元；建成工业项目58个，完成投资4.54亿元；区内实现国内生产总值13.54亿元，比上年增长28.5%；被命名为“全国乡镇企业科技园区”。

财政金融稳定运行。全市地方财政收入完成9.82亿元，比上年增长14.7%；财政支出20.55亿元，增长16.6 %。财政支出结构趋于优化，财政保障能力明显增强，保证了干部教师工资的发放和社会保障支出。金融机构各项存款余额 225.24亿元，比年初净增19.13亿元;各项贷款余额138.35亿元，减少3.65亿元。

社会事业全面发展。组织实施各类科技项目94项，推广重大科技成果15 项，新增民营科技企业38家。巩固提高“普九”成果，新创建规范化学校68所；全市普通高考被清华、北大、人大、科技大录取47人，连续3年居全省首位；濮阳职业技术学院开办启动工作稳步推进。计划生育工作进一步加强，人口自然增长率4.95‰。国有企业下岗职工基本生活费除应由企业承担部分外，做到了按时足额发放；企业离退休人员养老金按时足额发放；城市“低保”做到了应保尽保。全市新增就业岗位2.1万个，下岗职工再就业率47.3%。文化事业取得新成绩，成功举办第三届龙文化活动周，豫剧《村官》在全省巡演及晋京演出均获得成功，在第四届全国青少年杂技比赛中取得一金一银的佳绩。广播剧《村官李连成》等3件作品获省“五个一工程奖”。爱国卫生运动深入开展，城乡医疗卫生条件不断改善，市人民医院、市中医院荣获“全国百姓放心医院”称号，市卫生防疫站被评为“国家一等防疫站”。体育事业有新的发展，在省九运会上取得金牌34枚、总分997分，均比省八运会上升2个位次。城乡居民生活水平明显改善，城镇居民人均可支配收入5875元，比上年增长27 %；农民人均纯收入1948元，增长3.6%。土地管理、环境保护、民族宗教、外事侨务、安全生产、药品监督、质量技术监督、审计、统计、物价、民兵、史志、地震、气象、人防、残疾人事业等都取得了新的成绩。

精神文明和民主法制建设进一步加强。认真开展“三个代表”学教活动，进一步落实公民道德建设实施纲要，群众精神文化生活日益丰富，全市精神文明建设呈现新的局面。积极推进依法治市，广大干部群众的法制观念和政府部门的依法行政意识明显增强。清理建市以来市政府规范性文件和其他政策性文件4000多件，废止120件。自觉接受市人大及其常委会的依法监督，严格执行市人大及其常委会的决议。认真接受政协、民主党派、工商联的民主监督。按时办理人大代表建议129件、政协提案248件，满意率和基本满意率均在96%以上。坚持打防结合，落实社会治安综合治理各项措施，开展严打整治斗争，破获各类刑事案件4990起，摧毁各类犯罪团伙227个；有效打击了“法轮功”等邪教组织的犯罪活动，社会大局保持稳定。大力开展“双拥”活动，密切了军政、军民关系。全面落实党风廉政建设责任制，大力开展反腐败斗争。重视人民群众来信来访，妥善解决群众反映的问题。

各位代表，上述成绩的取得，是省委、省政府和市委正确领导的结果，是全市人民共同努力的结果。借此机会，我代表市人民政府向全市工人、农民、知识分子和干部，向驻濮部队、武警官兵、政法干警，向中央、省驻濮单位和社会各界人士表示衷心的感谢！

在肯定成绩的同时，也应清醒地看到，我市经济社会发展中还存在诸多问题。如城乡居民收入特别是农民收入水平偏低，农业抗灾能力不强，地方工业总量小，城镇化进程滞后，非公有制经济发展不足，企业环境有待进一步改善，金融机构存贷反差较大，社会保障和再就业任务繁重，地方财政困难等。对此，要在今后工作中着力加以解决。

二、2003年政府工作的总体要求和预期目标

今年是贯彻落实党的十六大精神、全面建设小康社会的第一年，做好今年的政府工作，保持经济社会发展的良好势头，具有十分重要的意义。围绕实现全面建设小康社会目标，到2020年，要在优化结构和提高效益的基础上，确保我市国内生产总值比2000年翻两番以上，人均国内生产总值高于全省平均水平，基本实现工业化。

今年政府工作的总体要求是：以邓小平理论和“三个代表”重要思想为指导，认真贯彻落实党的十六大和中央、省委经济工作会议精神，围绕全面建设小康社会的目标，深入实施“以工兴市” 战略，坚持“壮大实力抓工业，稳定基础抓农业，繁荣市场抓商贸，名城带动抓城建”的工作思路，以经济结构调整为主线，以改革开放和科技进步为动力，大力推进工业化和城镇化进程，全面繁荣农村经济，努力实现速度、质量、效益相统一，正确处理改革、发展、稳定的关系，推动社会全面进步，促进社会主义物质文明、政治文明、精神文明协调发展。

今年我市经济社会发展的主要预期目标是：国内生产总值增长10%左右，全社会固定资产投资增长10%左右，地方财政收入略高于经济增长，社会消费品零售总额增长10%，城镇居民人均可支配收入和农民人均纯收入分别增长8%和6%左右，人口自然增长率控制在7‰以内，城镇人口增加5万人，城镇登记失业率控制在4%以内。确定上述发展预期目标，既考虑了全面建设小康社会必须加快发展的要求，考虑了与“十五”计划的衔接，也考虑了支撑我市经济发展的现实环境、基础条件和各方面的承受能力，经过努力完全可以达到。

三、进一步扩大内需，促进经济持续快速增长

（一）打破交通瓶颈，构建经济发展的基础平台。一要加快濮鹤高速公路和汤台铁路技术改造工程建设，力争濮开高速公路、濮鹤高速连接线改城市道路等项目尽快开工，做好濮阳至菏泽高速公路规划。二要加强干线公路建设。投资1.35亿元，开工建设省道213线南乐吴家庄至清丰西阳邵段28.7公里改建工程；投资1.63亿元,开工建设106国道清河头至东明黄河桥段33.2公里改建工程。积极争取市西外环路和北环路、濮阳至柳屯至濮城公路尽快开工建设。加强县乡道路的改造和完善，加速城乡经济融合。

（二）加强重点项目建设，扩大固定资产投资。按照国家确定的资金投向和重点，选好项目，争取国债资金和中央、省各类资金的支持，扩大投资规模。今年全市着力抓产业和基础设施方面的113个重点项目，其中工业项目66个、农业项目9个、城建项目20个、交通项目8个、商贸项目10个。总投资91.08亿元，年内计划完成投资51.64亿元。要办好与全市人民群众生产、生活密切相关的16件实事：（1）筹资200万元，建成市社会保障和再就业服务中心，全市新增就业岗位2.8万个。（2）筹资1500万元，继续完善城区中小学教学设施。（3）筹资80万元，完善公安110指挥系统设施；筹资80万元，建设市120急救指挥中心，年内形成全市急救网络；筹资230万元，完善市185社会综合服务呼叫中心。（4）筹资300万元，完成黄河路西段、益民路等新建路段绿化和京开大道、人民路绿化改造任务。（5）筹资100万元，改造胜利小区、行政南小区和永安街等6条背街小巷。（6）筹资200万元，完成濮水河部分河道衬砌及调节池配套等设施建设。（7）筹资1000万元，完成市燃气扩建工程主干管网铺设。（8）筹资200万元，完善市体育场附属设施，确保“五一”投用。（9）筹资120万元，增加城市垃圾中转站和公厕。（10）筹资150万元，完成市老干部活动中心配套设施建设，上半年投用。（11）筹资500万元，基本建成市综合活动中心。（12）筹资1000万元，完成马颊河城区段河道综合治理工程，形成滨河风景带。（13）筹资1000万元，续建绿色庄园二期工程，“五一”试开业接待游客。（14）筹资600万元，完成西内环路拓宽改造。（15）筹资9100万元，完成农村电网改造二期工程；启动农村电网改造三期工程。（16）改善市民居住条件，年内新增经济适用住房面积10万平方米。在筹资问题上，要广开门路，特别要积极启动民间资金投入基础设施建设，凡是国家法律法规没有明令禁止的行业和部门，都要向民间资本开放；凡是对外资开放的投资领域，都要允许民间资本进入。市计委要定期发布鼓励民间资金投入的行业、产业和具体项目，各有关部门要加强服务和指导，最大限度地扩大民间投资。

（三）努力增加城乡居民特别是低收入者收入，增强消费动力。坚持多予、少取、放活的原则，促进农民增收。加强引导和服务，鼓励和组织农民外出务工，增加农民劳务收入。巩固和完善农村税费改革成果，确保农民负担不反弹。全面推行涉农价格收费公示制度，组织开展涉农收费专项治理。增加财政对“两个确保”、城市“低保”的投入，改善低收入者的生活水平。

（四）优化消费环境，培育和发展消费热点。彻底清理和取消各种限制消费的政策，完善鼓励和引导消费的各项政策措施。积极扩大住房、旅游休闲、教育培训、社区服务、文化娱乐、体育健身和卫生保健等领域的消费。普遍推行购房入户政策，规范房地产市场。发展经济适用住房和廉租房，满足低收入阶层住房需求。推进非义务教育发展，促进教育消费。完善商业和各种公益事业的服务网点，为拓宽城乡消费市场创造条件。

（五）做好财政金融工作，支持扩大内需。坚持依法治税，依率计征，加强征管，强化稽查，做到应收尽收。要垄断土地一级市场，在去年取得显著成效的基础上，加大土地使用权招标拍卖力度，对城市规划区内大型户外广告和群体户外广告设置权公开拍卖，拓宽经营城市的增收渠道。调整财政支出结构，优先安排工资发放和社会保障支出。严格控制和压缩一般性支出，除法律和政策规定需增加的重点支出外，其他各项支出实行零增长。牢固树立勤俭节约、艰苦奋斗的思想，坚决压缩会议、招待、出国考察等费用开支，严禁新开工建设脱离实际的“形象工程”、“政绩工程”。认真落实行政性收费和罚没收入“收支两条线”规定，加强预算管理，完善部门预算、国库集中收付和政府采购制度。

把扩大贷款规模作为今年金融工作的重点，充分发挥金融对扩大内需、促进地方经济发展的支持作用。积极向银行推荐有市场、有效益的项目，协调银行落实贷款承诺，支持重点企业和基础设施建设。扩大信贷投入，增加对结构调整、技术改造、高新技术项目、中小企业和民营企业的资金支持。扩大消费信贷，增强居民在住房、教育等方面的消费能力。开展创建金融安全区活动，优化金融信用环境，维护金融债权，防范化解金融风险。

四、围绕实施“以工兴市”战略，推进经济结构调整

（一）走新型工业化道路，加快工业化进程。工业化是实现现代化、全面建设小康社会的必由之路。要按照党的十六大确定的走新型工业化道路的要求，大力实施“以工兴市”战略，强力推进工业化进程。加快濮阳工业发展，必须在扩大增量和盘活存量上下功夫，重在扩大增量和提高效益。扩大增量就是项目拉动，要多上项目，上大项目，上效益好的项目，培育“双超”企业，扩大工业的规模和实力。要把石油化工、生物制品等技术密集型产业和农副产品加工、玻璃制品及其它劳动密集型产业作为发展重点，拉长产业链条，形成支柱产业。围绕这些支柱产业，培育一批市场竞争力强的骨干企业和主导产品。总投资14.34亿元的林纸一体化项目不仅是工业大项目，也是农业结构调整的龙头项目，要采取得力措施，确保年内开工建设。盘活存量就是搞活现有工业企业，使已有资产发挥效益。要坚定不移地支持中原油田、中原乙烯、中原大化、三强集团等大型企业的发展，年内中原油田投资17亿元，扩大原油、天然气生产能力；中原乙烯投资1亿元，5万吨混合碳四综合利用项目和10万吨芳烃抽提项目建成投产；中原大化开工建设三聚氰胺扩建项目，总投资4亿元；三强集团新建2×5万千瓦供热机组项目，总投资5亿元。要本着改造提升传统产业、做大做强新兴产业、培育发展高新技术产业的原则，构筑石油、化工化肥、玻璃制品、耐火材料、塑料编织和羽绒加工等产业，采取重点扶持、政策倾斜等措施，促其进一步膨胀规模。大力扶持市高新区和工贸示范区的生物化工园、医药工业园、文留特种玻璃工业园、柳屯塑编工业园，以及台前孙口羽绒工业园上规模、上水平。对产品质量低劣、浪费资源、污染严重的企业坚决关停，广泛推行清洁、文明生产方式。要抓住国际产业和国内沿海发达地区产业转移的先机，利用我市丰富的油气资源和城市效应，集中力量打好“城市牌”、“资源牌”，争取吸引大工业项目来濮落户，形成更大的聚集优势，做大做强板块经济。同时，大力发展民营工业，构建多元化工业发展格局。

（二）调整农村经济结构，全面发展农村经济。全面建设小康社会，重点在农村，难点也在农村。根本出路是推进农业结构战略性调整，实现产业化经营，推进农业现代化，促使农村经济由农业主导型向工业主导型转变，农村向城镇转变，农民向市民、工人、商人、民营企业家转变。这既是加快农村经济发展的根本措施，也是实施“以工兴市”战略的重要组成部分。要用发展工业的理念发展农业，实行动态管理，扶持重点骨干龙头企业壮大规模。以工贸小区建设为载体，引导乡镇企业向小城镇集中，城乡一体，工农并进，壮大县域经济。优质小麦和畜牧业发展，要更好地融入全省“两个基地”建设之中，争取省在资金、政策方面的扶持。按照省统一部署，搞好黄河滩区奶牛绿色养殖工程建设。继续抓好具有我市特色的优质稻米、优质果品、名优蔬菜、名优花卉、食用菌和波尔山羊、小尾寒羊等基地建设，形成一批专业大户、专业村、专业乡。实施“种子工程”，发展名、特、优、新、稀产品。强化农产品和畜产品的安全管理，抓好“菜篮子”工程。加强全市22个农业科技园区规范化建设，做好申报国家级农业高新技术园区工作。搞好农民技术培训，支持农村各类专业技术协会。推进农村基础设施和生态工程建设。争取金堤河治理二期工程立项，完成渠村灌区配套与节水改造工程，续建第二、第三濮清南配套工程，改造中低产田15万亩。完成19.2万亩退耕还林任务，抓好106国道改线工程等120公里绿色通道建设，力争实现全市平原绿化高级达标。完善兴农网，搞好农业信息服务。做好扶贫开发和以工代赈工作，把省政府关于农村“三有一不”政策落实情况列入县、乡政府的考核目标。按照省统一安排，尽快编制、实施黄河滩区农业综合开发规划。下大力气解决贫困人口的温饱问题，争取年内4万贫困人口实现脱贫。

（三）调整城乡结构，加快城镇化进程。坚持市城区、县城、小城镇三头并进的方针，着重抓好市城区和5个县城建设，提高对生产要素的聚集能力，培育特色支柱产业，以产业支撑和带动城镇化的迅速发展，促使农村剩余劳动力转移，提高非农人口的比重。市城区要巩固“四城三奖”的成果，以创建国际花园城市为动力，加快基础设施建设，拉大城市框架，扩大城市规模，完善城市功能。年内市东郊220千伏变电工程竣工，第二水源厂主体基本建成，新一代天气雷达系统主体竣工,加快国际会展中心建设。推进户籍管理制度改革，消除农民进城务工经商的壁垒。全面加强城市管理，治理卫生死角，清除占道经营，强化交通管理，提升城市品位。五个县城要按照濮阳市卫星城的目标，加快建设步伐，提高管理水平。充分发挥工贸示范区的示范带动作用，搞好10个重点镇建设。继续做好培养、建设100个西辛庄式社会主义新农村的工作。

（四）大力发展服务业，提高第三产业比重。积极筹集资金，加大旅游业投入，加强重点旅游项目建设，提高景区景点品位，扩大客源市场，促进旅游及其关联产业的发展。以建设三省交界流通中心为目标，做好规划论证和组织实施工作，建设巴黎商业街二期等专业批发市场。抓好商业中心、商业小区建设。推行连锁经营、物流配送、电子商务等流通方式，大力发展现代流通业。加强民间商会和行业协会建设，引导商户自我管理，严格自律，有序竞争。制定和实施城市社区服务业发展规划，加快家政服务、医疗保险等社区服务组织建设。发展和规范会计、法律、咨询等社会中介服务组织。

五、大力推进改革开放，为加快发展提供强大动力

（一）加大改革力度。按照建立现代企业制度的要求，加快国有企业规范的公司制改革和股份制改造，完善法人治理结构。争取年内市黄河化工厂等5户企业破产重组，中原啤酒厂等4户企业实行股份改造，市皮革厂等3户企业盘活资产，走出困境。采取更加灵活的方式，放开放活中小企业。国有企业改革必须从“改革的力度、发展的速度、社会承受的程度”三个方面统筹考虑，做到积极稳妥。依法稳定农村土地承包关系，纠正各种侵害农民土地承包权的违法行为。有条件的地方可按照依法自愿、有偿的原则进行土地承包经营权流转，逐步发展规模经营。继续推进粮棉流通体制改革，切实保护农民利益。要转变政府职能，改进管理方式，减少对市场主体的直接干预。按照省统一部署，积极推进事业单位管理体制改革。巩固和扩大行政审批制度改革成果，办好市行政服务中心。完善政府重大决策的规则和程序，推进决策科学化、民主化。

（二）扩大对外开放。认真组织参加中国（厦门）投资贸易洽谈会、中国（深圳）高新技术成果交易会等，提高参会成效。充分发挥驻外工作机构的作用，拓宽对外联系渠道。树立市外就是外的观念，采取重点招商、定向招商、以商招商等形式，大力引进外部资金、技术、人才和资源。农业方面，重点搞好“四引进”。即加快引进优质新品种，引进现代农业工程技术，引进农产品精深加工技术，引进农业标准化生产方式。工业方面，围绕“三个创新” 扩大引进。即通过引进促企业机制创新，带动我市企业建立符合市场经济要求的现代企业制度；通过引进促企业技术创新，利用成果转让、买断专利、技术入股、联合开发等形式，提高企业技术创新能力；通过引进促企业管理体制创新，使企业的计划、劳动、分配、财务、质量、营销等管理方式，逐步与国际惯例接轨。第三产业方面，支持外商独资、合资开发旅游资源，兴办旅游项目。积极引进外资改造国有商业，兴建大型专业批发市场和商业网点。实施多元化战略，扩大出口创汇。巩固现有出口市场，加大促销力度，提高市场占有率。积极开发新兴市场，着力扶持重点企业和重点出口产品，调整出口商品结构，加快由粗加工、低附加值向精加工、高附加值的转变升级。全市外贸出口总额增长12%左右。

（三）大力发展非公有制经济。非公有制经济是我市经济最具活力的增长点，要进一步放开、放手、放胆，清除各种障碍，落实扶持政策，使其在投融资、税收、土地使用等方面与公有制经济公平竞争，提高非公有制经济在国民经济中的比重。全面落实市委、市政府《关于大力发展个体私营经济的意见》，按照《濮阳市个体私营经济工作考核办法》，对县（区）、乡（镇）进行考核评比，表彰奖励发展个体私营经济的先进单位和先进个人。

（四）优化经济环境。要把优化经济环境，作为发展经济的一号工程来抓，严肃查处“三乱”行为，坚决打击“三强四霸”。加强舆论监督，对典型案件公开曝光。各执法执纪部门要文明执法，为企业发展提供优质服务。努力用良好的环境树形象，增信誉，促引进，促发展。进一步推进政务公开，推行首问负责、服务承诺、限时办结和失职追究制度，降低交易成本。积极推行“放水养鱼”的长期发展政策、让利生税的优惠招商政策，营造和谐向上的工作环境、优质高效的服务环境、文明祥和的社会环境、安全有序的法制环境，使濮阳成为吸引各种发展要素的“磁场”。坚持集中整治与制度建设相结合，突出重点，标本兼治，继续抓好整顿和规范市场经济秩序工作。严厉打击制售假冒伪劣商品的违法犯罪活动，从严查处破坏市场经济秩序的案件。高度重视信用环境和信用体系建设，打造诚信濮阳。广泛开展以诚信为主题的宣传教育活动，提高全市的诚信意识。加快建立企业和个人信用档案，逐步实施企业信用公示制。依法严惩不讲信用、破坏信用的行为。

六、发展各项社会事业

加强社会主义精神文明和民主法制建设

（一）大力实施科教兴濮战略，加快人才队伍建设。牢固树立科学技术是第一生产力的观念，强化科技的开发和应用。组织实施科技强工、科技富民和科技提升三大工程，重点开展新材料、生物技术、精细化工产品、农副产品精深加工等专项开发，培养市科技创新示范企业。把教育放在优先发展的战略地位，推进素质教育，提高教育质量和办学效益。加强基础教育，改善办学条件，年内新增“普九”巩固提高乡镇6个，创建市级规范化学校30所。积极发展职业教育和成人教育。强化高等教育，按照千亩校园、万人大学的标准，筹资1亿元，加快濮阳职业技术学院建设，年内达到部分新生入学条件。积极鼓励社会力量办学。坚持培养与引进相结合，加大人才、智力引进力度，重点引进高层次人才。要尊重知识，尊重人才，在全市形成人才脱颖而出、人尽其才的氛围，营造吸引人才、留住人才的良好环境。

（二）实施可持续发展战略，促进社会全面进步。全面落实《人口与计划生育法》，加强人口与计划生育工作，稳定低生育水平。合理开发、节约使用和保护水、土地、矿产等资源，提高资源利用效率。加强重点流域和行业的污染治理，改善城乡居民生活的环境质量。大力推进生态市建设，促进人与自然的和谐发展。要体现先进文化的要求，始终把社会效益放在首位，加强城乡文化建设，繁荣文学艺术。办好庆祝建市20周年、杂技艺术节等活动。重视文物的保护利用，积极申报国家历史文化名城。做好市电视编播大楼建设的前期工作。大力开展爱国卫生运动，改善城乡卫生条件，加强疾病控制和预防保健，提高医疗服务水平。加强药品的监督管理，确保人民群众用药安全。积极开展全民健身运动，增强人民体质，举办好第六届全市运动会。做好民族工作，促进各民族的团结。全面贯彻党的宗教政策，依法管理宗教事务，引导宗教与社会主义社会相适应。重视统计工作，加强对宏观经济运行的监测。加强国防教育，深入开展拥军优属和军民共建活动，推进城市民兵工作改革。继续做好审计、人事、外事侨务、广播电视、史志、地震、人防等工作。

（三）做好就业和社会保障工作。加强宏观调控和政策引导，把控制失业率、增加就业岗位纳入国民经济和社会发展计划。加快城市街道办事处、重点乡镇社会保障和劳动就业工作机构建设，打造基层劳动保障工作平台。认真落实下岗失业人员从事个体经营的优惠政策，为下岗失业人员自谋职业和自主创业提供小额贷款担保。大力发展商贸、旅游、餐饮、社区物业、托幼养老等行业，鼓励发展就业容量大的中小企业，积极发展具有比较优势的劳动密集型产业，广开就业门路。扩大对外劳务输出，开拓省际和国际劳务市场。按照“三三制”原则落实资金，确保国有企业下岗职工基本生活费和企业离退休人员养老金按时足额发放。规范城市“低保”工作，切实做到应保尽保。以个体、私营和外资企业为重点，扩大城镇养老、医疗、失业等社会保险覆盖面。

（四）加强社会主义精神文明和民主法制建设。深入进行 “三个代表”重要思想宣传教育，引导人们树立正确的世界观、人生观和价值观。贯彻公民道德实施纲要，广泛进行社会公德、职业道德和家庭美德教育，弘扬爱国主义精神，倡导尊老敬贤、见义勇为的良好风尚。继续开展各类文明创建活动，提高全民文明素质。反对封建迷信，破除陈规陋习，倡导文明健康的生活方式。

坚持依法治市，依法行政。加强法制宣传教育，提高公民法律素质。认真执行人大及其常委会的各项决议，自觉接受人大及其常委会的依法监督和工作监督，接受政协的民主监督。认真办理人大代表建议和政协委员提案。广泛听取各民主党派和工商联的意见，发挥工会、共青团、妇联等人民团体的桥梁纽带作用。依法保护妇女、儿童、残疾人和未成年人的合法权益。加强社会治安综合治理，严厉打击刑事犯罪和暴力犯罪，防范和惩治“法轮功”等邪教组织的犯罪活动。加大“扫黄打非”力度，净化社会风气。做好信访工作,及时化解矛盾，为改革和发展创造稳定的社会环境。

（五）加强政府自身建设，树立良好形象。各级政府要牢牢坚持以经济建设为中心，把发展这个第一要务抓紧抓好。要体察民情，了解民意，着力解决广大群众关心的热点、难点问题。开展以勤政建设为中心的效能监察活动，改进工作作风，提高工作质量和效率，做到令行禁止，政令畅通。全体政府工作人员必须牢固树立公仆意识，认真落实党风廉政建设责任制，勤奋学习，踏实工作，执政为民，不谋私利，造就一支政治合格、业务精良、作风正派、纪律严明的公务员队伍，不辜负全市人民的期望。

各位代表：

今年的工作要求高，任务重，让我们在省委、省政府和市委的领导下，高举邓小平理论伟大旗帜，全面贯彻“三个代表”重要思想，认真落实十六大精神，与时俱进，开拓创新，真抓实干，扎实工作，进一步开创我市现代化建设新局面。
